# Efficacy and safety of zilebesiran in adults with hypertension: a systematic review and meta-analysis of randomized controlled trials

**DOI:** 10.1007/s00228-026-04179-4

**Published:** 2026-09-16

**Authors:** Muhammad Hussain, Muhammad Hassan, Asharib Sohaib, Taha Ahmed, Saifullah Khan, Abdul Hannan, Hassan Abdul Aziz Dhedhi, Asma Naz, Ameer Haider Cheema, Faizan Ahmed

**Affiliations:** 1 Department of Medicine, Dow University of Health Sciences, Karachi, Pakistan; 2 University of Texas Southwestern, Dallas, USA; 3 Hackensack Meridian Health Jersey, Shore University Medical Center, Neptune, USA

**Keywords:** Zilebesiran, Hypertension, Blood pressure, RNA interference, Systematic review, Meta-analysis

## Abstract

**Background:**

Hypertension is a major modifiable risk factor for cardiovascular disease, yet global control rates remain low. Zilebesiran, an investigational RNA interference therapy, targets angiotensinogen to modulate the renin–angiotensin–aldosterone system. While previous reviews exist, updated evidence is needed to better characterize its efficacy and safety.

**Methods:**

A systematic search of PubMed, Cochrane Library, Google Scholar, Embase, and ClinicalTrials.gov was conducted using MeSH and free-text terms. Two reviewers independently extracted data and assessed risk of bias using RoB 2.0. A random-effects model was applied in RevMan 5.4, with heterogeneity evaluated using I² statistics.

**Results:**

Four studies involving 1,412 adults with mild to moderate hypertension were included. The primary outcome, placebo-adjusted change in office systolic blood pressure (SBP), showed a pooled mean difference (MD) of −7.16 mmHg (95% CI: −13.63 to −0.70). Secondary outcomes demonstrated significant reductions in 24-hour ambulatory SBP (MD: −9.92 mmHg; 95% CI: −18.74 to −1.10), daytime SBP (MD: −9.76 mmHg; 95% CI: −19.46 to −0.06), nighttime SBP (MD: −9.95 mmHg; 95% CI: −16.71 to −3.19), and 24-hour ambulatory diastolic blood pressure (DBP) (MD: −7.27 mmHg; 95% CI: −11.13 to −3.41). In subgroup analysis by follow-up duration, the reduction in daytime SBP was significantly greater at 3 months than at 6 months (*P* = 0.02), suggesting a potential attenuation of the antihypertensive effect over time. Zilebesiran markedly reduced angiotensinogen levels (MD: −1.00; 95% CI: −1.15 to −0.85). Overall adverse events were significantly more frequent with Zilebesiran compared with placebo (OR: 1.46), including increased risks of hyperkalemia and injection-site reactions, while serious adverse events did not differ significantly between groups.

**Conclusion:**

Overall, zilebesiran produced significant and consistent BP reductions, especially in ambulatory measures, with a favorable safety profile. However, evidence remains limited by small sample sizes and reliance on placebo comparators, and larger trials with active antihypertensive controls are needed to validate long-term efficacy and safety.

**Supplementary Information:**

The online version contains supplementary material available at https://doi.org/10.1007/s00228-026-04179-4.

## Abstract (highlights)

*****What is known about this research topic?***:**

- Hypertension is a highly prevalent condition and a major modifiable risk factor for cardiovascular disease, stroke, and chronic kidney disease.

- Despite the availability of multiple antihypertensive drug classes, global control rates remain low, highlighting the need for novel therapeutic options.

- Zilebesiran, an investigational small interfering RNA (siRNA), targets hepatic angiotensinogen production to suppress the renin-angiotensin-aldosterone system (RAAS) and lower blood pressure.

- Previous meta-analyses on Zilebesiran included only two randomized controlled trials with relatively small sample sizes, limiting the generalizability of findings.

*****What this study adds and future implications***:**

- Builds on previous meta-analyses that included only 2 RCTs; this updated analysis incorporates 4 RCTs (1,412 participants), including Kardia 3 and Desai 2025.
- Adds daytime and nighttime ambulatory BP measurements and diastolic BP analysis, not reported in previous meta-analyses.
- Applies more rigorous methodology, including GRADE assessment and risk-of-bias evaluation
- Confirms that Zilebesiran significantly reduces office and 24-hour ambulatory systolic and diastolic BP, with strongest effects in daytime and nighttime readings.
- Demonstrates plasma angiotensinogen reduction, supporting RAAS-suppressive mechanism.
- Expanded safety analysis: includes detailed assessment of adverse events (hyperkalemia, injection-site reactions) and serious events, which were not fully analyzed in previous meta-analyses, confirming overall tolerability.
- Exploratory subgroup analyses evaluated potential differences according to dose and treatment duration; however, these findings should be interpreted cautiously because of the limited number of contributing trials
- Supports Zilebesiran as a promising novel antihypertensive, highlighting need for larger, long-term trials with active comparators to assess durability, safety, and cardiovascular outcomes.

**Graphical Abstract:**

**Supplementary Information:**

The online version contains supplementary material available at https://doi.org/10.1007/s00228-026-04179-4.

## Graphical abstract

**Figure:**
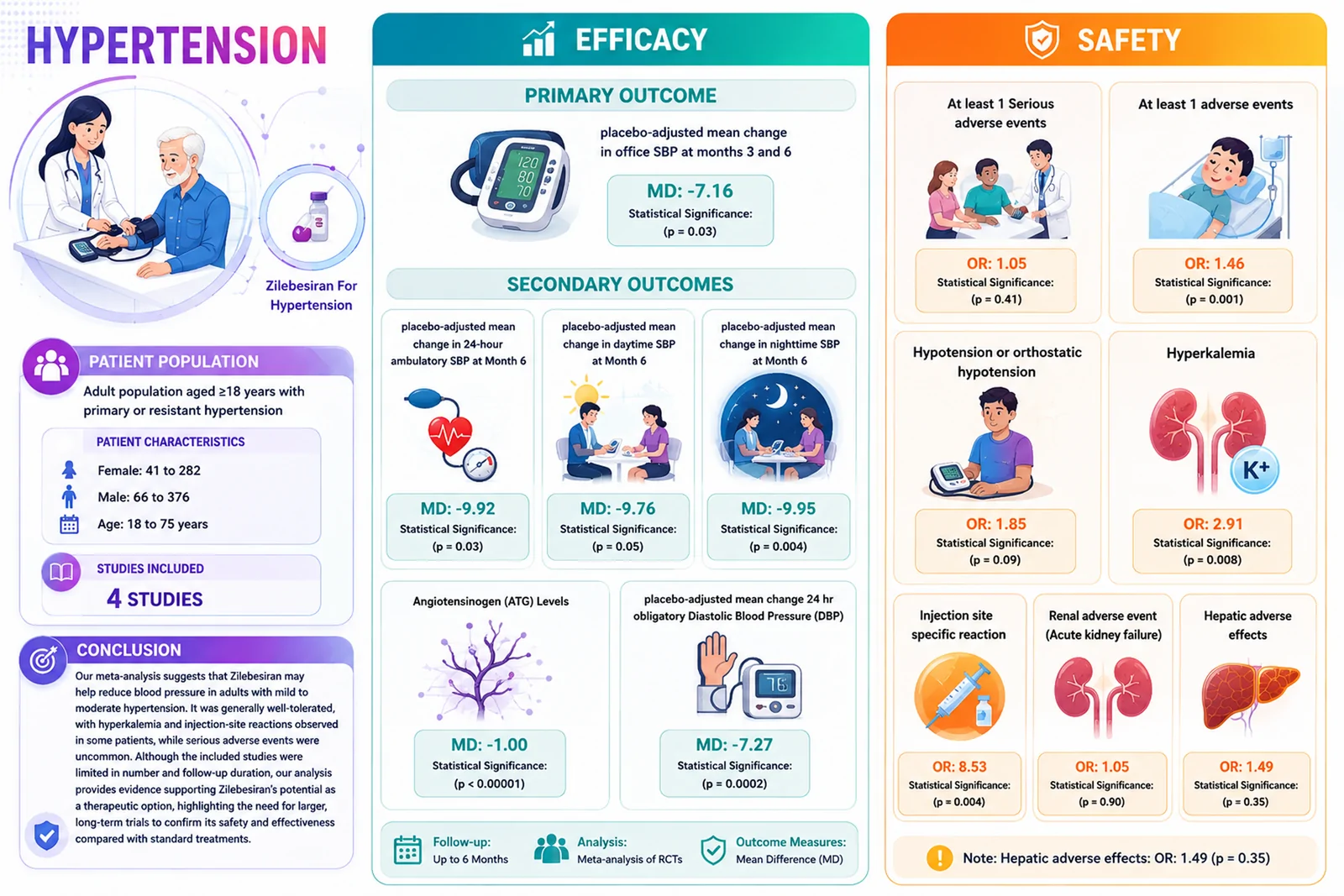

## Introduction

Hypertension is defined as a sustained elevation of blood pressure (BP) in the systemic circulation. Since various measurement techniques are used for the assessment of BP, diagnostic criteria have been set by the European Society of Hypertension (ESH) of ≥ 140/90 mm Hg, and the American College of Cardiology/American Heart Association (ACC/AHA) of ≥ 130/80 mm Hg [1], with further grades according to increased severity. Globally, nearly 1.4 billion people are affected by hypertension, which reflects its prevalence [2]. Furthermore, it is one of the leading modifiable risk factors for cardiovascular diseases (CVD), stroke, and chronic kidney disease [3, 4]. Given its multifactorial nature, with genetic, environmental, and lifestyle factors contributing to its development, effective management requires a comprehensive approach targeting all these determinants [5].

Hypertensive control is usually achieved through a combination of lifestyle modification and pharmacological interventions [6]. Antihypertensive drugs are a broad class of pharmacological agents that target different pathways to reduce BP. The various classes of drugs classified as antihypertensive include beta-blockers, angiotensin-receptor antagonists, angiotensin-converting enzyme inhibitors, renin inhibitors, calcium channel blockers, and diuretics [7]. However, despite the availability of these interventions, only 320 million (or 23%) adults have adequate control of hypertension [2]. This low control rate may be attributed to inadequate treatment, poor adherence, or resistant hypertension. Thus, the development and assessment of novel therapeutic options are also crucial to improving management.

Zilebesiran is one such investigational drug that is being developed to lower BP by targeting the renin-angiotensin–aldosterone system (RAAS), which has a central role in regulating BP. Zilebesiran is a small interfering RNA (siRNA) covalently linked to an N-acetylgalactosamine (GalNAc) ligand, which has high affinity for the hepatic asialoglycoprotein receptor [8]. This, in turn, reduces hepatic angiotensinogen mRNA levels, thereby lowering angiotensinogen levels [9]. Angiotensinogen is the precursor of angiotensin; decreased production results in suppression of RAAS activity and, in turn, reduced blood pressure.

Recent trials have shown promising results, but there is a substantial existing burden of hypertension, along with the presence of a significant knowledge gap regarding novel therapeutic options. Although previous meta-analyses and systematic reviews have been performed to address this topic, with the recent completion of additional trials, there is a need for an updated synthesis incorporating the newer evidence. An updated synthesis will better inform clinicians and policymakers to consider the efficacy and safety of this drug. To expand on the existing review, we conducted a systematic review and meta-analysis of published studies on Zilebesiran in patients with mild to moderate hypertension.

## Method

### Protocol registration

This systematic review and meta-analysis was conducted and reported in accordance with the Preferred Reporting Items for Systematic Reviews and Meta-Analyses (PRISMA 2020) guidelines. The protocol for this study has been registered on PROSPERO (CRD420251175365) [10].

## Data sources and search

A systematic literature search was conducted to identify relevant randomized clinical trials (RCTs) across PubMed, Cochrane Library, Google Scholar, Embase, and ClinicalTrials.gov, with studies published from inception to November 19, 2025, being included. The search strategy included combined Medical Subject Headings (MeSH) and free-text terms for “Zilebesiran”, “hypertension”, and “RCT”. The full search string is presented in Supplementary Table 1.

## Study eligibility criteria

### Data extraction and outcomes

Two independent reviewers extracted data from the included RCTs. Extracted data included basic study details (study name, clinical trial registration, DOI link, and treatment duration), baseline data (sample size, age, and sex), and details of the intervention and comparator (dosage and timing of Zilebesiran and placebo administration). To evaluate the safety and efficacy of Zilebesiran in patients with hypertension, the following outcomes were considered: the primary outcome was the mean change in office systolic blood pressure (SBP), whereas secondary outcomes included 24-hour ambulatory SBP, 24-hour ambulatory diastolic blood pressure (DBP), daytime SBP, nighttime SBP, and angiotensin levels. Similarly, adverse events included all adverse events, serious adverse events, hyperkalemia, injection-site reactions, hypotension or orthostatic hypotension, adverse events such as acute kidney failure, and hepatic adverse events. Additionally, subgroup analyses were performed based on treatment duration and dose to evaluate their influence on the estimated treatment effects. The included studies administered Zilebesiran subcutaneously as a single injection, using different dosing regimens across trials.

To address potential unit-of-analysis concerns, each randomized trial contributed only one comparison per outcome to the primary meta-analysis. When multiple doses or follow-up time points were available, the primary analysis used the 300 or 400 mg Zilebesiran dose at 6 months as the prespecified comparison. Placebo-adjusted treatment effects reported directly by the original studies were extracted whenever available. Multiple-dose and follow-up comparisons were evaluated only through exploratory subgroup analyses, including dose-based analyses at 6 months and treatment-duration analyses comparing 3-month versus 6-month follow-up. These subgroup comparisons were not considered independent observations in the primary pooled estimates.

### Statistical analysis

Statistical analyses were performed in Review Manager 5.4 using a random-effects model (DerSimonian and Laird) [11]. The included trials reported outcomes in different formats; continuous data was harmonized before pooling. Continuous outcomes were summarized as mean differences (MDs) with 95% confidence intervals. For studies providing placebo-adjusted mean differences with standard errors (SEs) or confidence intervals (CIs), effect estimates were entered directly using the generic inverse variance (GIV) method; CIs were converted to SEs using Cochrane-recommended formulas. For studies reporting pre- and post-treatment means and standard deviations (SDs), mean changes and SDs were calculated using the RevMan calculator. Dichotomous outcomes (e.g., ORR) were summarized as odds ratios (ORs) with 95% confidence intervals. Statistical significance was set at *p* < 0.05, and heterogeneity was assessed using the I² statistic (25%, 50%, and 75% representing low, moderate, and high heterogeneity) [12]. Publication bias was not assessed due to the limited number of studies (*n* = 4).

### Risk of bias assessment and sensitivity analysis

Two independent reviewers conducted risk of bias assessment for each included study using Cochrane Risk of Bias 2.0 (RoB 2.0) [13]. Each study was evaluated across five domains: randomization, blinding, completeness of outcome data, outcome measurement, and selective reporting. The overall risk of bias was categorized as low, some concerns, or high. A third independent reviewer resolved any disagreement between the two reviewers. Results were summarized using a risk-of-bias graph. Similarly, the certainty of evidence for all the outcomes was assessed using the Grading of Recommendations Assessment, Development and Evaluation (GRADE) approach. To evaluate the robustness of our findings, sensitivity analyses were performed to exclude the impact of bias and to identify which study disproportionately contributed to heterogeneity. Additionally, leave-one-out analysis was carried out to determine the effect of individual studies on the overall effect.

## Results

### Study selection

**Fig. 1:**
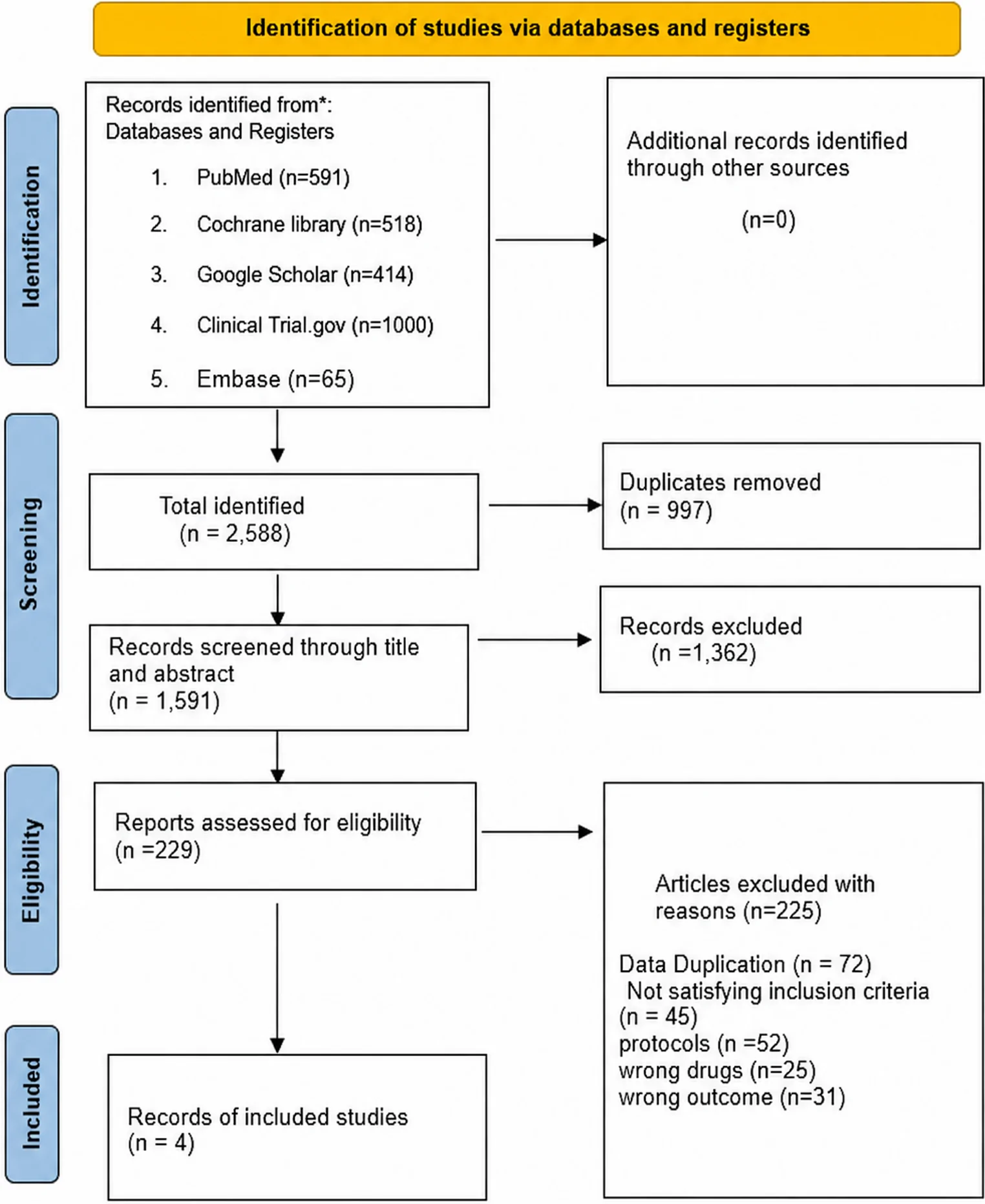
PRISMA flow chart

A total of 2,588 records were initially retrieved from five databases: PubMed, Cochrane Library, Google Scholar, ClinicalTrials.gov, and Embase. After removing 997 duplicates, 1,591 records were screened by title and abstract, leading to the exclusion of 1,362 references. The remaining 229 reports were assessed for eligibility through full-text review. A total of 225 articles were excluded due to reasons such as data duplication (*n* = 72), not satisfying inclusion criteria (*n* = 45), protocols (*n* = 52), wrong drugs (*n* = 25), and wrong outcomes (*n* = 31). Ultimately, four studies were included in the final analysis. The PRISMA flow chart summarizing the process is shown in Fig. 1.

### Study characteristics

Across the four included trials evaluating Zilebesiran, a total of 1,412 participants were enrolled, with individual study populations ranging from 107 to 658 participants. All trials evaluated subcutaneous administration of Zilebesiran using single-dose regimens, with background antihypertensive therapy permitted or administered in selected trials. The most frequently evaluated doses being 150 mg, 300 mg or 400 mg and 600 mg. One multicenter trial administered Zilebesiran in combination with standard antihypertensive agents such as indapamide, amlodipine, or olmesartan. All participants were adults aged 18 to 75 years, and sex distribution was generally balanced across trials, with male participants ranging from 66 to 376 and female participants from 41 to 282. The studies were conducted across multiple international locations, including the United States, United Kingdom, Canada, Estonia, Germany, Latvia, Lithuania, and Poland, with 4 to 110 participating centers. All included studies had a maximum follow-up duration of six months. Primary outcomes across trials focused consistently on reductions in 24-hour mean ambulatory systolic blood pressure or office systolic blood pressure (Table 1). (Fig. 8).

**Table 1:** Baseline characteristics of study populations included in the meta-analysis.

Study	Trial ID	Intervention	Dose	Route	*N* (Zilebesiran/Control)	Age	Sex (M/F)	Country	Follow-up	Primary Outcome
Bakris et al., 2024	NCT04936035	Zilebesiran	150, 300, 600 mg	SC	302/75	18–75 yr	210/167	USA, UK, Canada	6 months	24-h ambulatory SBP
Desai et al., 2023	NCT03934307	Zilebesiran	10–800 mg	SC	75/32	18–65 yr	66/41	UK	6 months	Adverse events
Desai et al., 2025	NCT05103332	Zilebesiran + standard therapy	600 mg + antihypertensive	SC	328/330	18–75 yr	376/282	USA, UK, Canada, Europe	6 months	24-h ambulatory SBP
Pagidipati et al., 2025	KARDIA-3	Zilebesiran	300 mg	SC	180/90	Median 67 yr	148/122	5 countries	6 months	Office SBP

### Effect of zilebesiran on mean change in office SBP

**Fig. 2:**
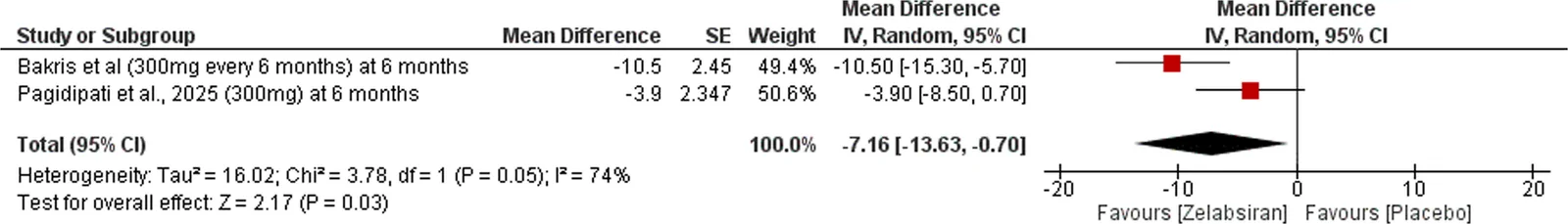
Forest plot of mean change in office systolic blood pressure (SBP) across included studies

The analysis found that Zilebesiran was associated with a significant reduction in office systolic blood pressure (SBP). The pooled mean difference (MD) for the mean change was (MD: −7.16 [−13.63, −0.70]; *p* = 0.03; I² = 74%) (Fig. 2). Subgroup analysis by time point did not reveal significant differences between groups (*P* = 0.49) (Supplementary Fig. 1). Subgroup analysis by dose did not reveal significant differences between groups (*P* = 0.92) (Supplementary Fig. 2).

### 24-Hour ambulatory SBP

**Fig. 3:**
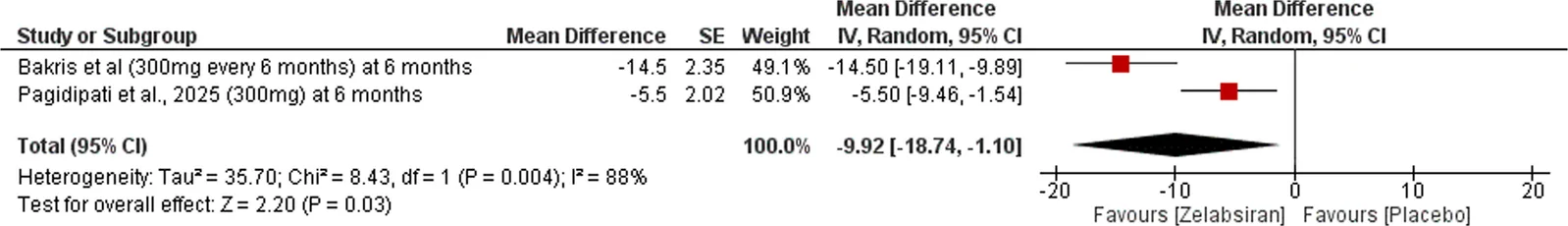
Forest plot of 24-hour ambulatory systolic blood pressure (SBP)

Across two primary studies, the analysis indicated that Zilebesiran was associated with a significant reduction in 24-hour ambulatory systolic blood pressure (SBP). The pooled mean difference (MD) for the mean change was (MD: −9.92 [−18.74, −1.10]; *p* = 0.03; I² = 88%) (Fig. 3). Subgroup analysis by time point showed a pooled mean difference of (MD: −10.73 [−12.83, −8.63]; *p* < 0.00001; I² = 72%), with no significant differences between groups (*P* = 0.62) (Supplementary Fig. 3). Similarly subgroup analysis by dose reveals no significant differences between groups (*P* = 0.33) (Supplementary Figs. 4).

### 24-Hour ambulatory daytime SBP

**Fig. 4:**
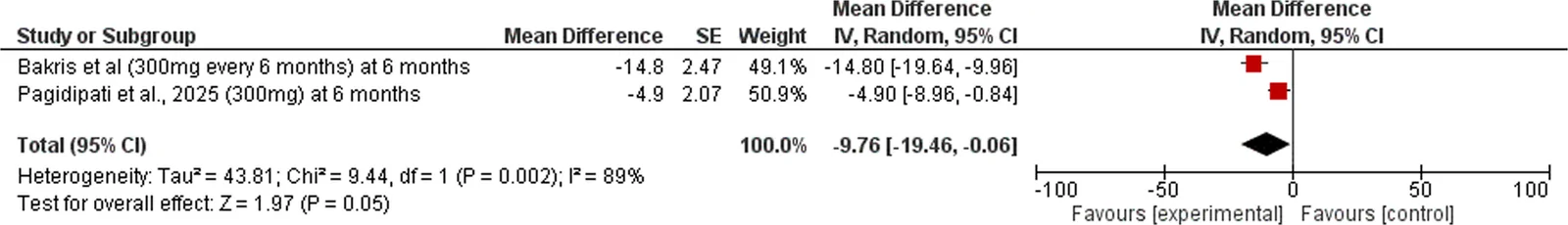
Forest plot of 24-hour ambulatory daytime systolic blood pressure (SBP)

The analysis, including two studies, revealed that Zilebesiran was associated with a significant reduction in 24-hour ambulatory daytime systolic blood pressure (SBP). The pooled mean difference (MD) for the mean change was (MD: −9.76 [−19.46, −0.06]; *P* = 0.05; I² = 89%) (Fig. 4). Subgroup analysis by follow-up time demonstrated reductions in daytime SBP at both 3 and 6 months; however, the magnitude of the treatment effect differed significantly between time points (*P* = 0.02), with a greater reduction observed at 3 months than at 6 months. This finding raises the possibility of attenuation of the antihypertensive effect over time. Subgroup analysis by dose demonstrated a pooled mean difference of (MD: −10.16 [−13.91, −6.42]; *P* < 0.00001; I² = 65%), with no significant differences between dose groups (*P* = 0.19) (Supplementary Figs. 5–6).

### 24-Hour ambulatory nighttime SBP

**Fig. 5:**
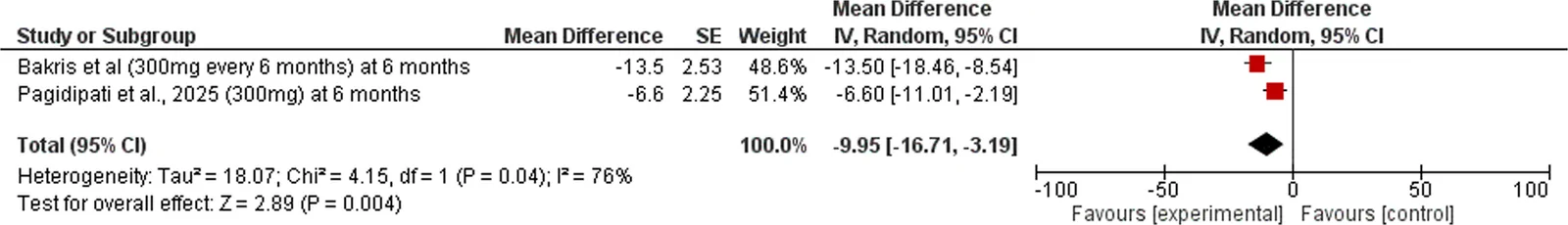
Forest plot of 24-hour ambulatory nighttime systolic blood pressure (SBP)

The analysis, which included two studies, showed that Zilebesiran was associated with a significant decrease in 24-hour ambulatory nighttime systolic blood pressure (SBP). The pooled mean difference (MD) for the mean change was (MD: −9.95 [−16.71, −3.19]; *p* = 0.004; I² = 76%) (Fig. 5). Subgroup analysis by time point did not indicate significant differences between groups (*P* = 0.10). Likewise, subgroup analysis by dose did not reveal significant differences between groups (*P* = 0.88) (Supplementary Figs. 7–8).

### Angiotensinogen (ATG) levels

**Fig. 6:**
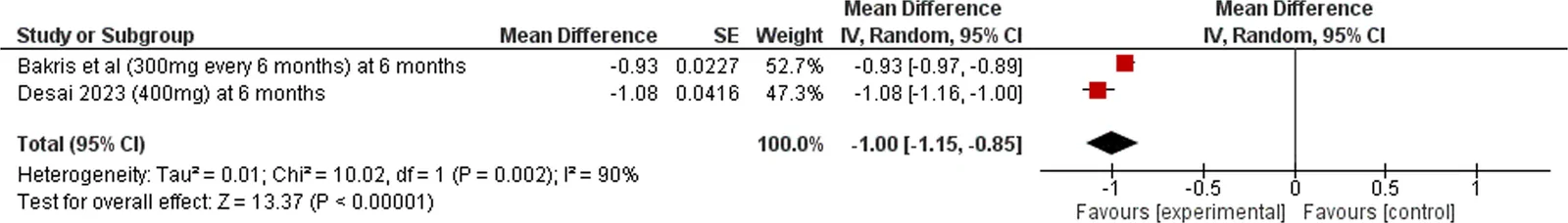
Forest plot of Angiotensinogen Levels (ATG)

Across the two primary studies, Zilebesiran was associated with a significant reduction in angiotensinogen (ATG) levels. The pooled mean difference (MD) for the mean change was (MD: −1.00 [−1.15, −0.85]; *p* < 0.00001; I² = 90%) (Fig. 6). Subgroup analysis by dose demonstrated a consistent reduction in ATG levels (MD: −0.99 [−1.06, −0.93]; *p* < 0.00001; I² = 89%), with no significant differences between groups (*P* = 0.15) (Supplementary Fig. 9).

### 24-Hour ambulatory diastolic blood pressure (DBP)

The analysis, based on two principal studies, revealed that Zilebesiran was associated with a significant reduction in diastolic blood pressure. The pooled mean difference (MD) for the mean change was (MD: −7.27 [−11.13, −3.41]; *p* = 0.0002; I² = 27%) (Supplementary Fig. 10). Subgroup analysis by time point showed a pooled mean difference of (MD: −8.26 [−9.14, −7.37]; *p* < 0.00001; I² = 0%), with no significant differences between groups (*P* = 0.39). Similarly, subgroup analysis by dose did not reveal significant differences between groups (*P* = 0.89) (Supplementary Figs. 11–12).

### Adverse effects

The pooled odds ratio for the occurrence of all adverse events associated with Zilebesiran was (OR: 1.46 [1.16, 1.85]; *P* = 0.001; I² = 0%) (Supplementary Fig. 13). The OR for serious adverse events was not statistically significant (*P* = 0.41) (Supplementary Fig. 14). A significant increase in the risk of adverse events was observed with Zilebesiran. Specifically, hyperkalemia (OR = 2.91, 95% CI: 1.32–6.42, *P* = 0.008, I² = 0%) (Supplementary Fig. 15) and injection-site reactions (OR = 8.53, 95% CI: 2.01–36.22, *P* = 0.004, I² = 0%) (Supplementary Fig. 16) occurred significantly more frequently in the Zilebesiran group compared with placebo. In contrast, no significant differences were noted for several other adverse outcomes, including hypotension or orthostatic hypotension (OR = 1.85, 95% CI: 0.90–3.79, *P* = 0.09, I² = 0%) (Supplementary Fig. 17), renal adverse events such as acute kidney failure (OR = 1.05, 95% CI: 0.52–2.09, *P* = 0.90, I² = 0%) (Supplementary Fig. 18), and hepatic adverse effects (OR = 1.49, 95% CI: 0.65–3.42, *P* = 0.35, I² = 0%) (Supplementary Fig. 19).

### Risk of bias assessment (ROB)

**Fig. 7:**
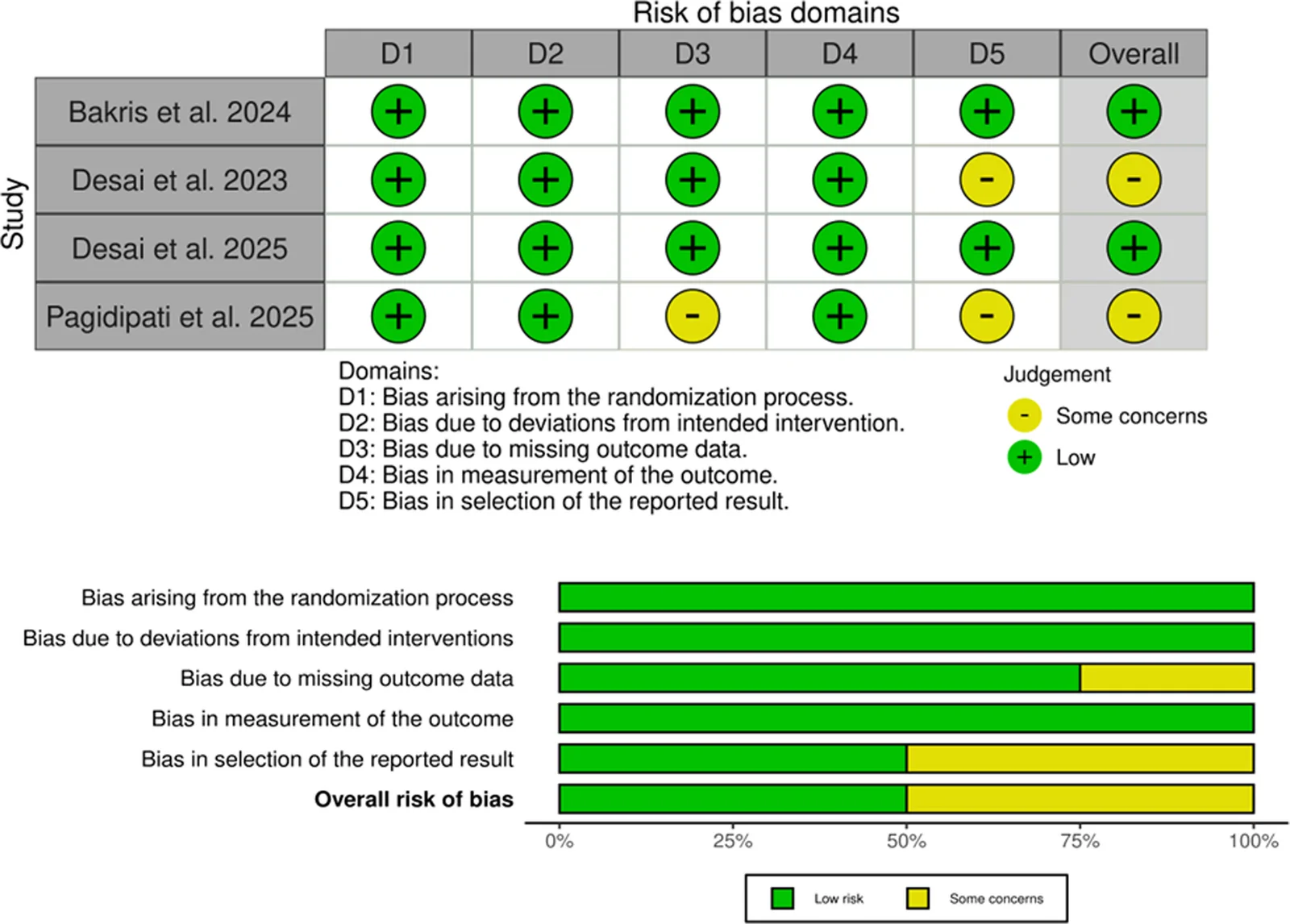
Risk-of-bias assessment for included studies reporting efficacy outcomes, evaluated across Cochrane RoB 2.0 domains

A total of four RCTs were included to assess the risk of bias regarding the efficacy of Zilebesiran. Two trials among these were judged to have a low risk of bias, which included Bakris et al. 2024 and Desai et al. 2025. However, there were some concerns in domain 5 concerning Desai et al. 2023 due to uncertainties about the selective reporting of efficacy outcomes. The fourth, Pagidipati et al. 2025, had some concerns in domains 3 and 5 due to the inadequacy of information for analysis of these domains, as the report was available in abstract form only (Fig. 7). For assessing the risk of bias regarding Zilebesiran’s safety, three of the reported trials were assessed as having a low risk of bias across all domains: Bakris et al. 2024, Desai et al. 2023, and Desai et al. 2025. However, in the Pagidipati et al. 2025 study, some concerns were noted in domains 3 and 5 because there was a lack of information about the completion of safety outcome data or pre-specified reporting, as the report was only in abstract form (Supplementary Fig. 20).

### Certainty of evidence

The certainty of evidence was assessed using the GRADE approach, considering risk of bias, inconsistency, indirectness, imprecision, and publication bias (Supplementary Table 2). The evidence supporting the blood pressure–lowering effects of Zilebesiran ranged from moderate to high certainty. The evidence for 24-hour ambulatory DBP was rated as high certainty due to consistent effects, low heterogeneity, and no serious concerns regarding imprecision. In contrast, the certainty of evidence for office SBP, 24-hour ambulatory SBP, and angiotensinogen (ATG) levels was rated as moderate certainty, primarily due to concerns regarding inconsistency arising from substantial heterogeneity and the limited number of contributing studies. The certainty of evidence for daytime SBP and nighttime SBP was rated as low certainty because of combined concerns related to substantial heterogeneity, limited contributing comparisons, and imprecision due to wide confidence intervals. The evidence for the overall incidence of at least one adverse event was rated as high certainty. However, the certainty of evidence for several safety outcomes was downgraded. Evidence for at least one serious adverse event and hyperkalemia was rated as moderate certainty due to serious concerns regarding imprecision, reflected by wide confidence intervals and limited event numbers. Furthermore, the evidence was rated as low certainty for hypotension/orthostatic hypotension, injection-site reactions, renal adverse events, and hepatic adverse events because of serious imprecision, with confidence intervals crossing the line of no effect or being substantially wide (Supplementary Table 2).

## Discussion

During this systematic review and meta-analysis, we found that Zilebesiran demonstrates robust antihypertensive efficacy across multiple blood pressure (BP) modalities. Compared to placebo, Zilebesiran is effective in reducing office SBP, 24-hour ambulatory SBP, 24-hour ambulatory daytime and nighttime SBP, angiotensinogen (ATG) levels, and 24-hour ambulatory diastolic blood pressure (DBP) in dose of 300 or 400 mg at six-month follow-up. These findings were consistent with previously published results from Bakris et al., Desai et al., and Pagidipati et al. [14–17].

Across all BP-lowering outcomes, Zilebesiran produced statistically significant reductions; however, the largest effect sizes were observed in ambulatory measurements. Ambulatory nighttime SBP demonstrated the largest reduction (−9.95 mmHg), followed closely by 24-hour ambulatory SBP (−9.92 mmHg) and daytime SBP (−9.76 mmHg), suggesting sustained blood pressure control throughout the day and night. This was followed by 24-hour ambulatory DBP (−7.27 mmHg), office SBP (−7.16 mmHg), and plasma ATG levels (−1.00). An important finding of this analysis was the significantly greater reduction in daytime SBP at 3 months compared with 6 months (*P* = 0.02). Although Zilebesiran continued to demonstrate a blood pressure–lowering effect at both time points, the attenuated treatment effect at 6 months raises the possibility of a reduction in antihypertensive efficacy over time. This finding is clinically relevant given the importance of sustained blood pressure control with long-acting RNA interference therapies. However, this observation should be interpreted cautiously, as the subgroup analysis was based on a limited number of comparisons, and differences in study populations, background antihypertensive therapy, and other study characteristics across follow-up periods may have contributed to the observed variation. Therefore, the available evidence cannot determine whether the smaller treatment effect at 6 months represents true attenuation of drug efficacy or between-study variability. Long-term randomized trials with serial assessments within the same study populations are needed to clarify the durability of Zilebesiran’s antihypertensive effect. Regarding safety, Zilebesiran was not associated with a significant increase in serious adverse events compared with placebo; however, the pooled analysis demonstrated a higher incidence of overall adverse events, hyperkalemia, and injection-site reactions. These findings indicate the need for continued safety monitoring, particularly for electrolyte abnormalities and injection-site reactions. Importantly, because the evidence base is currently limited to phase I and II trials, further studies involving larger and more diverse populations will be essential to fully characterize the long-term safety profile and detect less common adverse reactions.

The marked difference in the relative magnitude of ambulatory versus office SBP reductions between KARDIA-1 (Bakris et al.) and the studies by Desai et al. warrants consideration. In KARDIA-1, the greater reduction observed in ambulatory SBP compared with office SBP may partly reflect the study design, in which 24-hour ambulatory SBP served as the primary efficacy endpoint and eligibility was based on daytime ambulatory SBP following washout of background antihypertensive therapy. Thus, ambulatory BP measurements were central to both patient selection and efficacy assessment and were obtained repeatedly using standardized 24-hour monitoring [16]. In contrast, KARDIA-2 (Desai et al., 2025) evaluated zilebesiran as add-on therapy following a standardized antihypertensive run-in [15], whereas the earlier phase 1 study by Desai et al. (2023) evaluated BP outcomes as exploratory endpoints under additional dietary sodium and irbesartan coadministration conditions [14]. These differences in study design, treatment setting, patient selection, background therapy, and endpoint hierarchy may have contributed to the contrasting patterns observed across trials. Although repeated office BP measurements may attenuate the white-coat effect and potentially yield greater apparent reductions in office BP over time, this phenomenon alone does not fully account for the observed discrepancy. Because the individual trials were not designed to directly compare office and ambulatory BP responses, the precise explanation for these differences remains uncertain.

Moderate to high heterogeneity was observed in certain analyses, likely reflecting differences in study design, baseline characteristics, background therapy, dosing schedules, and follow-up durations across trials. However, interpretation of heterogeneity should be approached cautiously, particularly for outcomes with a limited number of contributing comparisons, as the reliability of heterogeneity estimates may be affected by the small evidence base. Compared with previous meta-analyses, the present study incorporates an additional RCT (Desai et al., 2025) instead of a previously published poster (Saxena et al.) and an additional poster dataset (Pagidipati et al.), while also expanding the range of evaluated outcomes beyond office SBP and 24-hour SBP to include daytime/nighttime ambulatory SBP, ambulatory DBP, and ATG levels [15, 17–19]. This provides a more comprehensive assessment of the anti-hypertensive potential of Zilebesiran. Chronic low-grade inflammation has emerged as an important contributor to the pathophysiology of hypertension, with inflammatory mediators such as C-reactive protein, interleukin-6, and tumor necrosis factor-α contributing to endothelial dysfunction, oxidative stress, vascular remodeling, and sustained blood pressure elevation. Since Zilebesiran suppresses hepatic angiotensinogen synthesis and reduces downstream renin–angiotensin–aldosterone system activity, it is biologically plausible that modulation of this pathway may influence inflammation-related processes. However, direct clinical evidence demonstrating reductions in inflammatory biomarkers with Zilebesiran is currently unavailable, and the present meta-analysis did not evaluate inflammatory outcomes. Therefore, any potential anti-inflammatory effects of Zilebesiran remain hypothetical and require confirmation in future studies incorporating dedicated inflammatory biomarker assessments [20]. Similarly, recent systematic review by Raza et al. reported significant reductions in blood pressure with zilebesiran across the available randomized controlled trials, with no significant differences in major safety outcomes [21]. Our findings are broadly consistent with these results and further characterize the efficacy and safety of zilebesiran. In addition, our analysis focuses on the 300 or 400-mg dose at 6 months and incorporates more studies with treatment-setting subgroup analyses, providing a complementary assessment of the evolving randomized evidence.

### Future recommendations

Future trials should analyze head-to-head comparisons with standard therapies, comparing Zilebesiran with established antihypertensive agents to better contextualize its clinical value. Additionally, long-term outcome trials (MACE, stroke, HF) should also be evaluated. Larger, more diverse patient populations are needed to assess variability in treatment response across age groups, ethnicities, and comorbidity profiles. Identifying response predictors should be a focus of future studies to support personalized dosing and improve outcomes. These findings not only validate Zilebesiran’s novel mechanism of action but also point to opportunities for refining its clinical use.

### Limitations

This meta-analysis has several important limitations that should be acknowledged. First, the number of eligible studies was limited, which reduces the statistical power and restricts the generalizability of the findings. Furthermore, one trial contributed a substantial proportion of the overall sample, which may have disproportionately influenced the pooled estimates. Second, substantial heterogeneity was observed in several key outcomes, including office SBP, 24-hour ambulatory daytime SBP, nighttime SBP, and angiotensinogen (ATG) levels. This variability likely reflects differences in study design, baseline characteristics, background antihypertensive therapy, dosing schedules, and follow-up durations across trials. Differences in treatment setting and background antihypertensive therapy may also affect safety outcomes, particularly hyperkalemia and acute kidney injury; therefore, pooled estimates may not fully capture treatment-specific safety signals. Third, all included studies were phase I or phase II trials, with follow-up periods extending to a maximum of six months. As a result, long-term efficacy, durability of BP reduction, and the potential for late-emerging adverse effects remain unknown. Furthermore, these early-phase trials enrolled relatively small and often homogeneous populations, limiting the applicability of findings to broader, more diverse patient groups. Lastly, the meta-analysis primarily relied on placebo-controlled comparisons, rather than direct comparisons with guideline-recommended antihypertensive therapies. Consequently, the relative safety and effectiveness of Zilebesiran against standard treatments (e.g., ACE inhibitors, ARBs, diuretics, or calcium channel blockers) cannot be determined from the available evidence. The restriction to English-language publications may have introduced potential language bias and may have resulted in omission of relevant studies published in other languages. The apparent attenuation of the daytime SBP reduction at 6 months compared with 3 months also warrants cautious interpretation, as differences in the number of contributing comparisons and study characteristics across follow-up time points may contribute to the observed variation. Consequently, the available data are insufficient to determine whether this represents true attenuation of the antihypertensive effect or between-study variability.

A further limitation relates to the handling of multiple comparisons from individual trials. To minimize potential double-counting and avoid inflation of precision, the primary analyses included only one prespecified comparison per randomized trial, using the 300 or 400 mg Zilebesiran dose at 6 months when multiple doses or follow-up time points were available. Placebo-adjusted treatment effects reported directly by the original studies were used whenever available. However, exploratory subgroup analyses evaluating different doses at 6 months and treatment-duration comparisons between 3 and 6 months may still involve correlated comparisons derived from the same trial. These subgroup findings were therefore interpreted cautiously and should be further evaluated in future analyses using approaches such as multilevel meta-analysis or robust variance estimation as additional trials become available. Additionally, the limited number of independent studies precluded meaningful meta-regression analyses to explore potential sources of heterogeneity. Therefore, potential effect modifiers require confirmation in future studies with larger sample sizes. Additionally, although inflammation may contribute to hypertension pathogenesis and RAAS activation, inflammatory biomarkers were not assessed in the included trials; therefore, this meta-analysis cannot determine whether Zilebesiran exerts direct anti-inflammatory effects.

## Conclusion

Our meta-analysis suggests that Zilebesiran may help reduce blood pressure in adults with mild to moderate hypertension. While serious adverse events were not significantly increased compared with placebo, Zilebesiran was associated with higher rates of overall adverse events, hyperkalemia, and injection-site reactions, emphasizing the need for continued safety monitoring. Although the included studies were limited in number and follow-up duration, our findings support the potential therapeutic role of Zilebesiran while highlighting the need for larger, long-term trials to further establish its safety profile and comparative effectiveness against standard antihypertensive therapies.

## Supplementary Information

Below is the link to the electronic supplementary material.

Supplementary Material 1 (DOCX 1.07 MB)

## Data Availability

All data analyzed in this study are publicly available from the included randomized controlled trials. References and supplementary materials provide full access to the datasets used.

## Declarations

### Clinical trial number

Not aaplicable.
