# Supplementary material for: Efficacy and safety of zilebesiran in adults with hypertension: a systematic review and meta-analysis of randomized controlled trials
Source: Eur J Clin Pharmacol. 2026 Sep 16;82(10):252. doi: 10.1007/s00228-026-04179-4 (PMC13582068; doi:10.1007/s00228-026-04179-4)
Supplement: Supplementary file 1 [file 228_2026_4179_MOESM1_ESM.docx]

**Supplementary Files :**

**Supplementary Figure 1.** Subgroup analysis of office systolic blood pressure (SBP) by time point.

**Supplementary Figure 2.** Subgroup analysis of office systolic blood pressure (SBP) by Dose.

**Supplementary Figure 3.** Subgroup analysis of 24-hour ambulatory systolic blood pressure (SBP) by time point.

**Supplementary Figure 4.** Subgroup analysis of 24-hour ambulatory systolic blood pressure (SBP) by dose.

**Supplementary Figure 5.** Subgroup analysis of 24-hour ambulatory daytime systolic blood pressure (SBP) by time point.

**Supplementary Figure 6.** Subgroup analysis of 24-hour ambulatory daytime systolic blood pressure (SBP) by dose.

**Supplementary Figure 7.** Subgroup analysis of 24-hour ambulatory nighttime systolic blood pressure (SBP) by time point.

**Supplementary Figure 8.** Subgroup analysis of 24-hour ambulatory nighttime systolic blood pressure (SBP) by dose.

**Supplementary Figure 9.** Subgroup analysis of angiotensinogen (ATG) levels by dose.

**Supplementary Figure 10.** Forest plot of 24-hour diastolic blood pressure (DBP).

**Supplementary Figure 11.** Subgroup analysis of 24-hour ambulatory DBP by time point

**Supplementary Figure 12.** Subgroup analysis of 24-hour ambulatory DBP by dose.

**Supplementary Figure 13.** Forest plot of odds ratios for all adverse events.

**Supplementary Figure 14.** Forest plot of odds ratios for serious adverse events.

**Supplementary Figure 15.** Forest plot of odds ratios for hyperkalemia.

**Supplementary Figure 16.** Forest plot of odds ratios for injection-site reactions.

**I****Supplementary Figure 17.** Forest plot of odds ratios for hypotension or orthostatic hypotension.

**Supplementary Figure 18.** Forest plot of odds ratios for renal adverse events.

**Supplementary Figure 19.** Forest plot of odds ratios for hepatic adverse events.

**Supplementary Figure 20.** Risk-of-bias assessment for included studies reporting safety outcomes evaluated across Cochrane RoB 2.0 domains.

| **Database** | **Date searched** | **Search strategy (exact syntax)** | **Records retrieved** |
| --- | --- | --- | --- |
| **PubMed** | 19 November 2025 | (("Zilebesiran" OR "ALN-AGT01" OR "AD-85481" OR "siRNA therapy" OR "RNA interference") AND ("Hypertension"[Mesh] OR hypertension OR "high blood pressure" OR "elevated blood pressure" OR "arterial hypertension")) | 591 |
| **Cochrane Library** | 19 November 2025 | ("Zilebesiran" OR "ALN-AGT01" OR "AD-85481" OR siRNA OR "small interfering RNA" OR "RNA interference") AND (hypertension OR "high blood pressure" OR "arterial hypertension" OR "elevated blood pressure") | 518 |
| **Google Scholar** | 19 November 2025 | ("Zilebesiran" OR "ALN-AGT01" OR "AD-85481") AND (hypertension OR "high blood pressure") | 414 |
| **[ClinicalTrials.gov](http://clinicaltrials.gov)** | 19 November 2025 | ("Zilebesiran" OR "ALN-AGT01" OR "AD-85481") AND (hypertension OR "high blood pressure") | 1000 |
| **Embase** | 19 November 2025 | ('hypertension'/exp OR hypertension:ti,ab OR "high blood pressure":ti,ab) AND (zilebesiran:ti,ab,kw OR "ALN-AGT01":ti,ab,kw OR "AD-85481":ti,ab,kw) | 65 |

**Supplementary Table 1.** Database-specific search strategies and number of records retrieved for zilebesiran in hypertension (searched on November 19, 2025)

| **Outcome** | **No. of participants (studies)** | **Effect estimate (95% CI)** | **Risk of Bias** | **Inconsistency** | **Indirectness** | **Imprecision** | **Publication Bias** | **Certainty of Evidence** |
| --- | --- | --- | --- | --- | --- | --- | --- | --- |
| **Office SBP (Placebo-adjusted mean change)** | 2 studies | MD -7.16 [-13.63, -0.70] | Not serious | Serious (I² = 74%) | Not serious | Not serious | N/A | Moderate |
| **24-hour Ambulatory SBP** | 2 studies | MD -9.92 [-18.74, -1.10] | Not serious | Serious (I² = 88%) | Not serious | Not serious | N/A | Moderate |
| **24-hour Ambulatory Daytime SBP** | 2 studies | MD -9.76 [-19.46, -0.06] | Not serious | Serious (I² = 89%) | Not serious | Serious (wide CI) | N/A | Low |
| **24-hour Ambulatory Nighttime SBP** | 2 studies | MD -9.95 [-16.71, -3.19] | Not serious | Serious (I² = 76%) | Not serious | Serious (wide CI) | N/A | Low |
| **Angiotensinogen (ATG) Levels** | 2 studies | MD -1.00 [-1.15, -0.85] | Not serious | Serious (I² = 90%) | Not serious | Not serious | N/A | Moderate |
| **24-hour Ambulatory DBP** | 2 studies | MD -7.27 [-11.13, -3.41] | Not serious | Not serious (I² = 27%) | Not serious | Not serious | N/A | High |
| **At least 1 Serious Adverse Event** | 3 studies | OR 0.76 [0.40, 1.46] | Not serious | Not serious | Not serious | Serious (wide CI) | N/A | Moderate |
| **At least 1 Adverse Event** | 3 studies | OR 1.46 [1.16, 1.85] | Not serious | Not serious (I² = 0%) | Not serious | Not serious | N/A | High |
| **Hypotension / Orthostatic Hypotension** | 3 studies | OR 1.85 [0.90, 3.79] | Not serious | Not serious (I² = 0%) | Not serious | Serious (CI includes no effect) | N/A | Low |
| **Hyperkalemia** | 3 studies | OR 2.91 [1.32, 6.42] | Not serious | Not serious (I² = 0%) | Not serious | Serious (wide CI, few events) | N/A | Moderate |
| **Injection-Site Reaction** | 3 studies | OR 8.53 [2.01, 36.22] | Not serious | Not serious (I² = 0%) | Not serious | Serious (very wide CI) | N/A | Low |
| **Renal Adverse Event (Acute Kidney Failure)** | 3 studies | OR 1.05 [0.52, 2.09] | Not serious | Not serious (I² = 0%) | Not serious | Serious (CI crosses no effect) | N/A | Low |
| **Hepatic Adverse Event** | 3 studies | OR 1.49 [0.65, 3.42] | Not serious | Not serious (I² = 0%) | Not serious | Serious (CI crosses no effect) | N/A | Low |

**Supplementary Table 2.** GRADE summary of efficacy and safety outcomes for zilebesiran. Effect estimates are shown as mean differences (MD) or odds ratios (OR) with 95% confidence intervals. Evidence quality was assessed across risk of bias, inconsistency, indirectness, imprecision, and publication bias, and rated as high, moderate, or low.

| **Study** | **Group / Subgroup** | **Dosage** | **Duration** | **Reduction in Office SBP (mmHg)** | **Reduction in 24-hr Ambulatory SBP (mmHg)** |
| --- | --- | --- | --- | --- | --- |
| **Pagidipati et al., 2025** | Zilebesiran 300 mg | 300 mg | 3 months | −5.0 ± 2.47 | −3.6 ± 2.07 |
| **Pagidipati et al., 2025** | Zilebesiran 600 mg | 600 mg | 3 months | −3.3 ± 2.50 | −2.6 ± 2.13 |
| **Pagidipati et al., 2025** | Zilebesiran 300 mg | 300 mg | 6 months | −3.9 ± 2.35 | −5.5 ± 2.02 |
| **Pagidipati et al., 2025** | Zilebesiran 600 mg | 600 mg | 6 months | −3.6 ± 2.35 | −7.4 ± 2.02 |
| **Bakris et al., 2024** | Zilebesiran 150 mg q6mo | 150 mg every 6 months | 3 months | −9.6 ± 2.17 | −14.1 ± 2.60 |
| **Bakris et al., 2024** | Zilebesiran 300 mg q3/6mo | 300 mg every 3 or 6 months | 3 months | −12.0 ± 1.89 | −16.7 ± 2.27 |
| **Bakris et al., 2024** | Zilebesiran 600 mg q6mo | 600 mg every 6 months | 3 months | −9.1 ± 2.19 | −15.7 ± 2.60 |
| **Bakris et al., 2024** | Zilebesiran 150 mg q6mo | 150 mg every 6 months | 6 months | −7.5 ± 2.47 | −11.1 ± 2.40 |
| **Bakris et al., 2024** | Zilebesiran 300 mg q6mo | 300 mg every 6 months | 6 months | −10.5 ± 2.45 | −14.5 ± 2.35 |
| **Bakris et al., 2024** | Zilebesiran 300 mg q3mo | 300 mg every 3 months | 6 months | −12.1 ± 2.58 | −14.1 ± 2.42 |
| **Bakris et al., 2024** | Zilebesiran 600 mg q6mo | 600 mg every 6 months | 6 months | −10.2 ± 2.19 | −14.2 ± 2.40 |
| **Desai et al., 2023** | Zilebesiran 200 mg | 200 mg | 3 months | NR | −14.3 ± 16.3* |
| **Desai et al., 2023** | Zilebesiran 400 mg | 400 mg | 3 months | NR | −12.6 ± 2.8 |
| **Desai et al., 2023** | Zilebesiran 800 mg | 800 mg | 3 months | NR | −16.2 ± 3.2 |
| **Desai et al., 2025** | Zilebesiran 600 mg + indapamide | 600 mg (+ indapamide) | 3 months | −18.5 ± 2.19 | −12.1 ± 2.27 |
| **Desai et al., 2025** | Zilebesiran 600 mg + amlodipine | 600 mg (+ amlodipine) | 3 months | −10.2 ± 1.66 | −9.7 ± 1.61 |
| **Desai et al., 2025** | Zilebesiran 600 mg + olmesartan | 600 mg (+ olmesartan) | 3 months | −6.7 ± 1.76 | −4.5 ± 1.89 |
| **Desai et al., 2025** | Zilebesiran 600 mg + indapamide | 600 mg (+ indapamide) | 6 months | −13.6 ± 1.68 | −11.0 ± 1.89 |
| **Desai et al., 2025** | Zilebesiran 600 mg + amlodipine | 600 mg (+ amlodipine) | 6 months | −8.6 ± 1.17 | −7.9 ± 1.35 |
| **Desai et al., 2025** | Zilebesiran 600 mg + olmesartan | 600 mg (+ olmesartan) | 6 months | −4.5 ± 1.15 | −1.8 ± 1.43 |

**Supplementary Table 3.** Placebo-adjusted change in office and 24-hour ambulatory systolic blood pressure across included trials, by dose group and follow-up time point. Values represent mean placebo-adjusted change from baseline ± standard error (mmHg). SBP, systolic blood pressure; NR, not reported.
